# Editorial: 10th anniversary of Frontiers in Surgery - celebrating progress and envisioning the future of multidisciplinary surgery

**DOI:** 10.3389/fsurg.2026.1866857

**Published:** 2026-06-23

**Authors:** Dennis P. Orgill, Rei Ogawa, Gabriel Sandblom, Stefano Cianci, Małgorzata Wierzbicka, Jaimo Ahn, Stavros K. Kakkos

**Affiliations:** 1 Harvard Medical School, Boston, MA, United States; 2 Nippon Medical School, Tokyo, Japan; 3 Department of Clinical Science and Education Södersjukhuset, Karolinska Institutet, Solna, Sweden; 4 Unit of Gynecology and Obstetrics, Department of Human Pathology of Adult and Childhood “G. Barresi”, University of Messina, Messina, Italy; 5 Faculty of Medicine Wroclaw University of Science and Technology, Wrocław, Poland; 6 Grady Memorial Hospital/Emory University, Atlanta, GA, United States; 7 Department of Vascular Surgery, University of Patras, Patras, Greece

**Keywords:** artificial inteligence, patient safety, surgery, surgical (technical), technology

## Introduction

**Figure 1:**
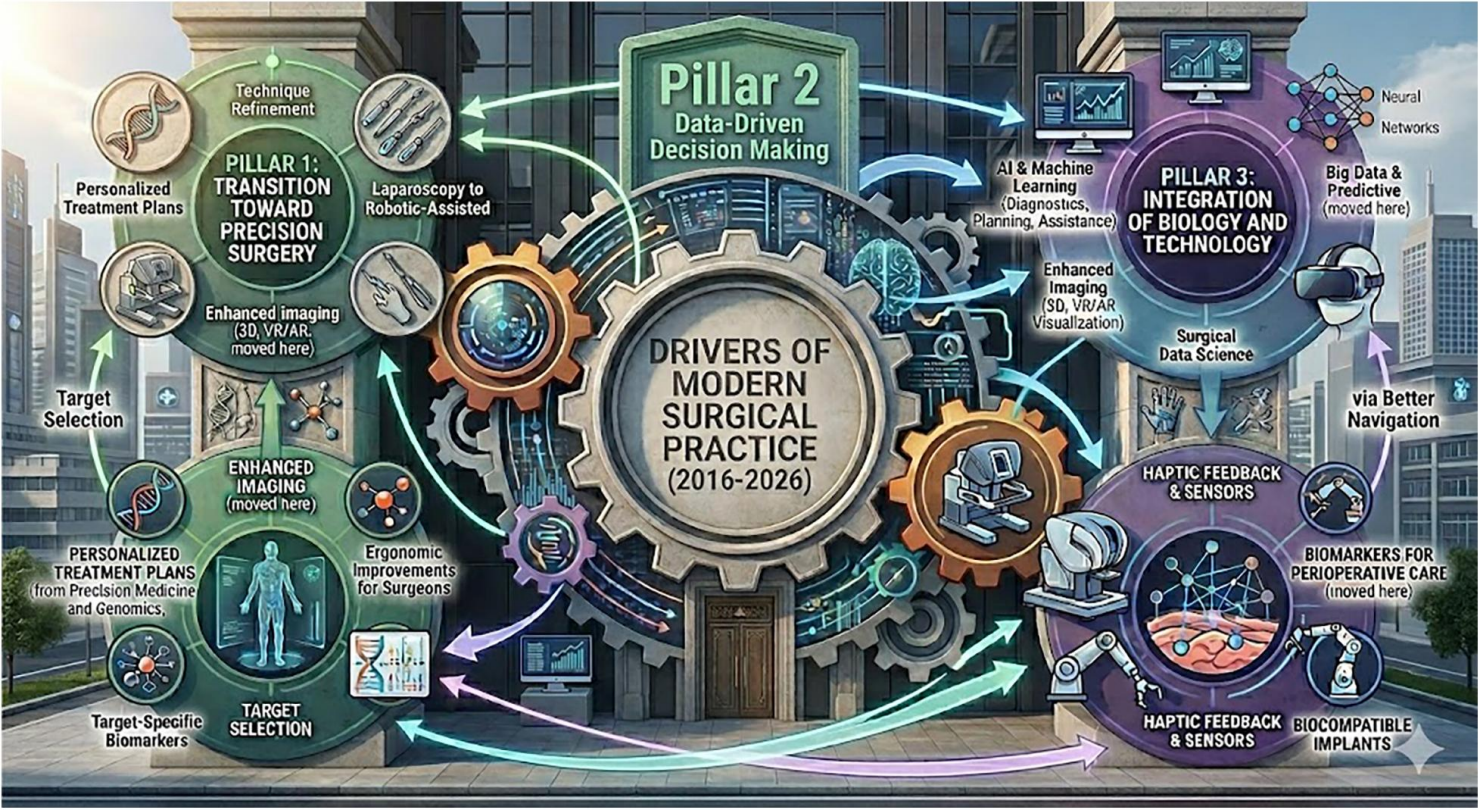
AI-generated conceptual diagram (gemini) illustrating three cross-cutting pillars of surgical advancement over the last 10 years. This figure is intended to show the power of AI in concept visualization. This is an illustrative synthesis based on the text of this editorial, generated by Gemini (May 3, 2026).

**Figure 2:**
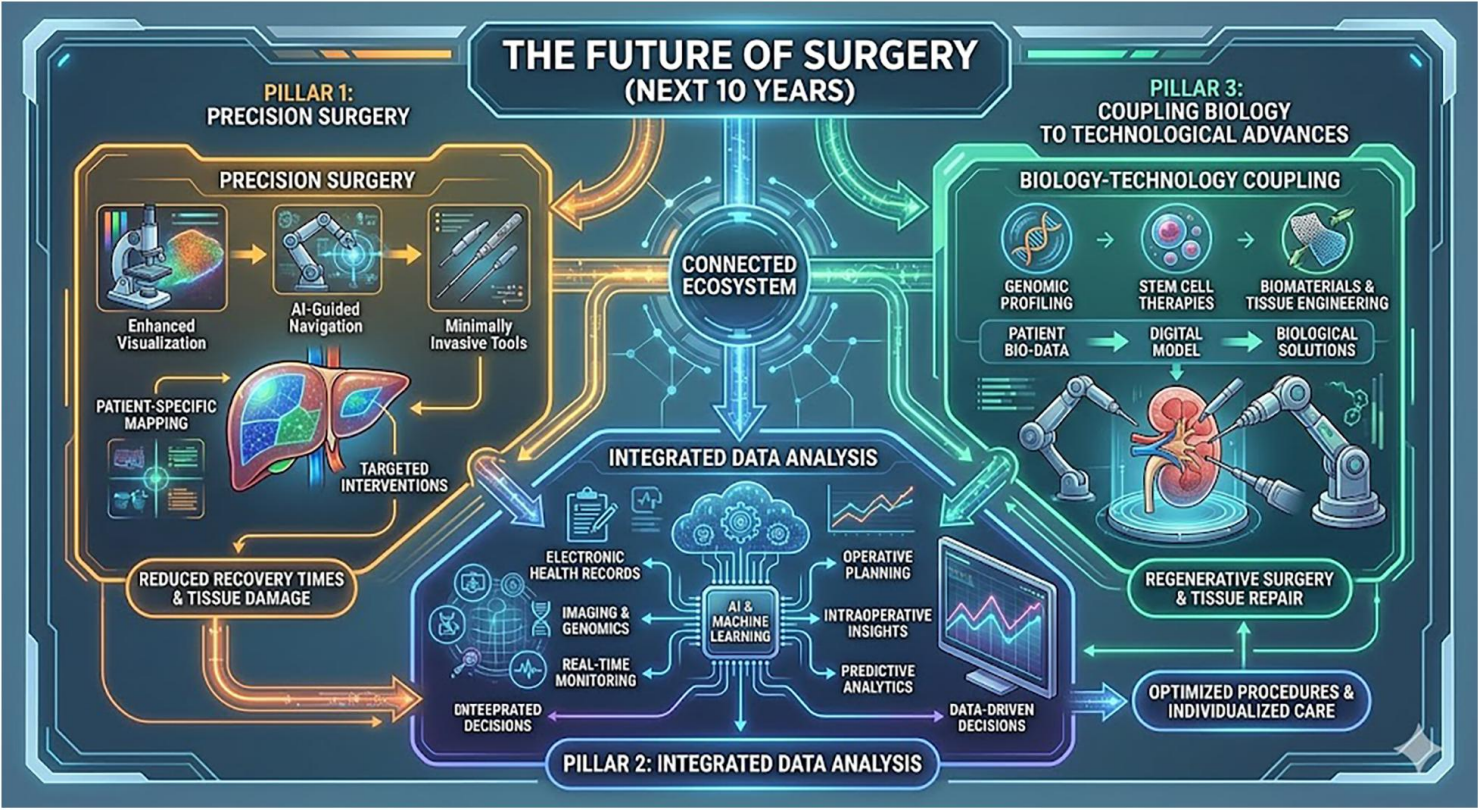
AI generated image (gemini) for the future of surgery over the next 10 years. This is an AI generation based on our paper that points out in graphic form some possible future directions.

The 10th Anniversary of *Frontiers in Surgery* offers an opportunity to reflect on how surgery has changed—not only through incremental technical advances, but through several broad paradigm shifts that now cut across nearly every sub-specialty. To date (4/15/2026), 6,659 papers have been published. Over this period, surgical progress has been built on 3 overall pillars: (1) Precision surgery, (2) data-driven decision making, and (3) integration of biology with technology. Precision surgery is best reflected in the boom in robotics but also in better visualization through microscopes, 3D printing, navigational systems and improved imaging. Data-driven decision making has allowed surgeons to better analyze their data, more efficient literature reviews and quality improvement initiatives which allow us to better counsel our patients on the pros and cons of proposed surgical procedures, as well as emerging AI-enabled decision support. While subspecialties have advanced along different trajectories, these common themes have repeatedly enabled safer operations, better selection of patients and procedures, faster recovery, and improved long-term outcomes. In this anniversary editorial, we highlight both the broad advances as well as specific advances in subspecialties and make some predictions of how we may view an exciting future. This list is clearly not comprehensive but shows how each of these domains is present in the sub-specialties reviewed (Figures 1, 2).

### Reconstructive and plastic surgery

#### Precision surgery

Microsurgery has become safer and more reliable, with further improvements achieved through the refinement of supermicrosurgical techniques, perforator flap surgery, lymphovenous anastomosis, and enhanced perioperative management. The integration of three-dimensional imaging, virtual surgical planning, and patient-specific devices has improved precision in craniofacial surgery, breast reconstruction, and complex reconstructive procedures.

In aesthetic surgery, the development of minimally invasive techniques and energy-based devices has improved safety and postoperative recovery. Collectively, these advances demonstrate the evolution of plastic and reconstructive surgery toward more precise, biologically grounded, and patient-centered care.

#### Data-Driven decision making

Large database retrospective studies have provided longitudinal data which synthesizes the risks of procedures, providing clinicians with real world data to advise patients. These data have been important in areas of informing patients of risks of rare tumors associated with breast implants.

#### Integration of biology and technology

The field of regenerative medicine has also advanced significantly, with expanded clinical applications of biomaterials and tissue-engineering strategies. At the same time, deeper insights into mechanobiology and wound healing have fundamentally transformed the concept of scar management. In particular, the treatment of keloids and hypertrophic scars has progressed dramatically, with the establishment of multimodal strategies that combine surgery, radiotherapy, pharmacologic therapy, and tension-reduction techniques based on biomechanical principles. Furthermore, the use of high-resolution imaging and molecular profiling has enhanced our understanding of the pathophysiology of pathological scars, paving the way for more targeted therapies.

### Visceral surgery

Over the last 10 years, substantial improvements have been seen across various aspects of visceral surgery. Although the ultimate goals remain unchanged, whether to restore the integrity of the abdominal wall, remove a sick organ, or monitor an intestinal obstruction, the measures taken to achieve these objectives have undergone great development.

### Precision surgery

The development of robot-assisted laparoscopy has also become routine in bariatric surgery. Various strategies have been suggested to modify intestinal anatomy, thereby managing obesity and its metabolic effects. In a pilot study of two cases using robot-assisted laparoscopy, severe obesity with type 2 diabetes mellitus was managed with ileal interposition, duodenal closure, and sleeve gastrectomy (Filho et al.). By also performing sympathectomy during the same procedure, autonomic dysfunction could, at least in theory, improve peripheral perfusion and insulin sensitivity. The study may show the way for future studies that incorporate the anatomical advantages of robot-assisted surgery with improved understanding of metabolism in obesity and diabetes.

### Data-Driven decision making

The basic principles of open and laparoscopic groin hernia repair, including the anatomical positioning of the mesh, have changed little in the last thirty years. By applying the principles of enhanced recovery after surgery, the safety of emergency hernia surgery and postoperative recovery have been improved (Zhuet et al.). Such advances may be achieved without introducing new technology or acquiring advanced resources.

The long-term outcome after treatment of pancreatic cancer remains poor, despite great efforts to detect the tumors at an early stage and attempts to improve oncological treatment. The short-term outcome may, however, be improved if carried out under epidural anesthesia. A study from Dubai showed that this is feasible if the patients are well informed and adequately selected (Hassan et al.).

#### Integration of biology and technology

The laparoscopic technique has revolutionized gallstone surgery during the last three decades. Visualization of bile ducts, however, has remained a great challenge. Using indocyanine green fluorescence, bile ducts may also be visualized in cases of extensive local inflammation. In a cohort study on patients undergoing laparoscopic cholecystectomy with fundus first approach, it was found that the use of indocyanine green fluorescence is feasible and safe (Haverinen et al.).

### Obstetrics and gynecological surgery

#### Precision surgery

Modern gynecological surgery is undergoing a transformative shift toward ultra-minimally invasive surgery (UMIS) (1) and data-driven approaches. UMIS utilizes micro-instruments with a diameter as small as 2 mm to 3 mm, effectively performing complex laparoscopies through incisions the size of a needle. This approach, often referred to as “needlescopic surgery,” results in negligible scarring and a significant reduction in post-operative analgesia requirements. Parallelly, Single-Port Laparoscopy (or LESS - Laparoendoscopic Single-Site Surgery) consolidates all surgical access into a single 1.5 cm incision, typically hidden within the umbilicus. A major innovation is the rise of VNOTES (Vaginal Natural Orifice Transluminal Endoscopic Surgery), which allows surgeons to perform procedures like hysterectomies through the vaginal canal, leaving no visible abdominal scars and significantly reducing recovery time (2).

#### Data-driven decision making

Robotic-assisted platforms are evolving with the integration of Artificial Intelligence (AI) to provide real-time intraoperative decision support, such as identifying tissue characteristics and vascular distributions to enhance surgical precision. Next-generation systems, like the Medtronic Hugo™, are expanding the accessibility of complex robotic procedures (3) meanwhile the introduction of the da Vinci Single Port (SP) system has addressed previous ergonomic challenges by providing 360-degree range of motion through one entry point.

#### Integration of biology and technology

Non-incisional treatments like the Sonata procedure, which utilize ultrasound-guided radiofrequency energy to treat fibroids, epitomize the trend toward preserving uterine integrity while minimizing patient trauma. These advancements collectively aim to shift major surgeries into the outpatient setting, prioritizing faster return to daily activities and improved quality of life.

### Otorhinolaryngology—head & neck surgery

The Head & Neck section of Frontiers in Surgery has illuminated a series of converging advances that have reshaped oncologic, functional, and organizational paradigms in head and neck surgery. (Frontiers in Surgery, Otorhinolaryngology – Head and Neck Surgery section overview). At the conceptual level, a decisive achievement has been the translation of Donabedian's classic triad of structure, process, and outcome into a contemporary definition of “best practice” tailored to malignant tumors of the upper aerodigestive tract. (Licitra, G., 2020) This modern framework emphasizes not only technical excellence in the operating theatre, but also the environment in which surgery is delivered, the robustness of care pathways, and the systematic auditing of results over time (Hassan et al.).

### Precision surgery

Parallel to this structural transformation, the last decade has witnessed a profound revolution in surgical access, dominated by the rise and refinement of minimally invasive transoral and endoscopic techniques (Licitra G. 2020) (Hassan et al.; Haverinen et al.; Filho et al.; 1). Transoral laser microsurgery (TLM) has evolved from a niche modality into a widely accepted organ-preserving approach for selected tumors of the oropharynx, hypopharynx, and larynx, permitting piecemeal resections with exquisite margin control and, in experienced hands, oncologic outcomes that rival those of traditional open procedures (Hassan et al.). Transoral robotic surgery (TORS), initially confined to early HPV-related oropharyngeal cancers following regulatory approval, has progressively expanded its indications to encompass more challenging subsites, including the supraglottic larynx, hypopharynx, and parapharyngeal space, always under the admonition that adoption must be evidence-driven and tempered by rigorous patient selection (Hassan et al.; Filho et al.; 1). The shared thread uniting these approaches is the deliberate retreat from morbid external incisions and mandibulotomies, in favor of routes that preserve external appearance, shorten hospitalization, and expedite functional recovery, all while maintaining an uncompromising commitment to negative surgical margins (Haverinen et al.; Filho et al.).

Reconstructive surgery has undergone its own quiet revolution, driven by the convergence of microvascular technique, perforator flap design, and enhanced recovery principles (Hassan et al.). Free tissue transfer is now firmly established as the standard of care for reconstruction of complex composite defects of the oral cavity, pharynx, and facial skeleton, with large national series documenting high flap survival rates and increasingly sophisticated strategies for salvage in the rare event of failure (Hassan et al.) The evolution toward perforator flaps has, in turn, allowed surgeons to minimize donor-site morbidity and to tailor the tissue envelope with greater precision to the three-dimensional requirements of the defect, thereby improving both functional outcomes, particularly speech and swallowing, and aesthetic integration (Hassan et al.). These technical refinements are closely intertwined with the implementation of Enhanced Recovery After Surgery (ERAS) protocols specifically designed for major head and neck procedures involving free flaps (Hassan et al.). Such pathways, grounded in evidence-based perioperative care, have reduced complication rates, shortened hospital stays, and optimized resource utilization, without eroding the complexity or ambition of the reconstructive effort (Hassan et al.).

### Data-driven decision making

Centralization of care in high-volume centers and in the hands of high-volume surgeons has emerged as a consistent determinant of improved survival and reduced perioperative morbidity in head and neck squamous cell carcinoma, reinforcing the notion that complex oncologic surgery should be concentrated in specialized units with mature multidisciplinary infrastructures (Hassan et al.).

#### Integration of biology and technology

A comparable shift has occurred in the management of sinonasal malignancies and anterior skull base tumors, where endoscopic techniques have enabled an increasingly “histology-driven” strategy (Hassan et al.). Carefully selected histologic entities, those with favorable patterns of local spread and predictable response to adjuvant therapy, may now be addressed via purely endonasal or combined endoscopic approaches, with complete radiologic resection (gross total resection) and negative margins as explicit goals (Hassan et al.). These developments have forced the community to revisit seemingly settled questions, such as the precise definition of a “clear margin” in the context of piecemeal or endoscopic resections and the acceptable incidence and management of cerebrospinal fluid leaks as a quality metric for skull base surgery (Hassan et al.). The discourse has thus moved beyond feasibility to a sophisticated debate on standards, benchmarks, and the limits of minimal invasiveness in an anatomically constrained region.

Equally transformative has been the maturation of neck management as a cornerstone of curative treatment, rather than a secondary adjunct to resection of the primary tumor (Hassan et al.). High-level evidence, now broadly disseminated, has definitively established elective neck dissection in early T1N0 oral cavity cancer as superior to a “wait-and-see” policy, conferring gains in overall and disease-free survival by pre-emptively addressing occult nodal disease (Hassan et al.). At the same time, the Robbins classification of neck dissections has achieved near-universal adoption as a common language, enabling precise communication of the extent and intent of cervical surgery across institutions and studies (Hassan et al.). Within this framework, sentinel lymph node biopsy in cN0 oral cavity cancer has emerged as one of the most consequential minimally invasive innovations: by accurately staging the neck with minimal morbidity, it offers a means to spare selected patients from the functional and cosmetic burden of more extensive dissections without compromising oncologic safety (Hassan et al.).

Finally, perhaps the most far-reaching change chronicled in recent years is the integration of surgical innovation into broader oncologic and systemic trends, namely de-escalation, personalization, and relentless quality oversight. The advent of HPV-related oropharyngeal carcinoma, with its distinctly favorable prognosis, has catalyzed a wave of de-escalation trials that seek to reduce treatment-related toxicity while preserving cure rates, whether by attenuating the intensity of CRT or by leveraging minimally invasive surgery as the principal modality in selected, well-stratified cohorts (Hassan et al.; Haverinen et al.). At the same time, the growing armamentarium of systemic therapies, targeted agents, immunotherapy, and adaptive radiotherapy, has further underscored the centrality of complete surgical resection (R0) and appropriate neck control as the foundation upon which sophisticated multimodal strategies can be constructed. Parallel advances in diagnostics, including narrow-band imaging, autofluorescence, high-resolution imaging, and emergent liquid biopsy technologies, have begun to influence both the selection of candidates for conservative surgery and the early detection of local recurrence, thereby tightening the feedback loop between diagnosis, treatment, and surveillance. The cumulative effect of these intertwined developments is a discipline that has deliberately moved from the “art of large operations” to a rigorously standardized, outcomes-driven surgical oncology, one that aspires to reconcile maximal oncologic radicality with minimal invasiveness and the best possible restoration of function.

### Orthopedic surgery

#### Precision surgery

There continues to be an evolution in orthopedic implants and medical devices which are now commonly customized for an individual patient. Many of these can be 3-D printed in advance for use by the surgeon. 3-D cutting guides can also be useful in doing tumor resections.

#### Data-driven decision making

Orthopedic conditions limit the lives of most adults with an estimated 30 million orthopedic procedures performed annually. In the US, 1 in 3 Americans report a musculoskeletal condition; they may be suffering from chronic back pain, have been involved in a motor vehicle related trauma or have passed away after sustaining a geriatric fragility hip fracture. The greatest global advancement we have made against orthopedic problems may indeed be the combination of recognition of the burden of disease (https://www.ors.org/boneandjointburden/) and international efforts—through surgeons, physicians, health providers, scientists, engineers, concerned citizens, policy-makers and patients—to improve the musculoskeletal human condition every day. There are certainly important themes to be found: we create greater surgical impact with smaller surgical footprints, more and more using technology such as image guidance and robot assistance; our diagnostics are safer and more accurate.

#### Integration of biology and technology

Biologics are not only emerging but more on-target; our clinical studies are better executed and more applicable to the patient right in front of us; our shared patient conversations continue to be refined and electronic health records nudge us toward better decision-making beyond the “burden” they can be perceived to be. Perhaps one day, our policy makers, health providers and consumers will all see as one.

### Vascular surgery

#### Precision surgery

Vascular surgery has demonstrated enormous progress during the last decade.

Standard endovascular aneurysm repair (EVAR) has been revolutionized with the use of dedicated endografts dealing with hostile neck anatomy, including short and conical necks, obviating the need for open repair; the latter is associated with a higher mortality and morbidity rate, while long term results up to 10 years and beyond will be required to assess the durability of this novel EVAR approach for patients with an abdominal aortic aneurysm (AAA).

Endovascular repair of complex AAAs and thoracoabdominal aortic aneurysms has also advanced, leading to a much safer repair for a challenging patient group, where open repair has had a high mortality rate, even in selected patients. The move towards the thoracic aorta has gone past the left subclavian and now includes the aortic arch and the ascending aorta.

#### Data-driven decision making

The long-term data found in many of our databases guides to better decisions particularly relating to endograft type and open vs. minimally invasive techniques. In wound healing, there have been a large number of studies focusing on biological, synthetic and semi-synthetic devices to facilitate healing in patients with difficult-to-heal wounds.

#### Integration of biology and technology

Peripheral arterial disease has seen a surge of endovascular solutions, such as use of drug eluting ballons and bioabsorbable stents. The vascular community has realized that long term cardiovascular mortality of patients with arterial and aneurysmal disease is the most significant threat, related to risk factors that include smoking and dyslipidemia; the latter is managed with statins, but the combination with PCSK9 pathway inhibitors, such as alirocumab, inclisiran and the awaiting FDA approval oral enlicitide, to achieve LDL targets has revolutionized patient care.

### Future directions

Past events are not a great predictor of the future, but they are the best we currently have. Major scientific breakthroughs, geopolitical conflicts and economic disruptions could dramatically shape the future. For example, the emergency of GLP-1 drugs and the dramatic changes in several surgical fields could not have been precisely predicted 10 years ago. We are hopeful that other major breakthroughs in fields such as aging and longevity could cause paradigm shifts in how we approach surgical patients.

We expect that improvements in technology will simplify surgery and take away some of the mundane tasks that surgeons now perform while improving the safety of our patients. We expect widespread adoption of AI to help surgeons with documentation and will also prompt surgeons to perform diagnostic testing or manage pharmaceuticals which are becoming increasingly complex. Having AI review the chart to look for rare drug interactions or propose rare disease states will be a great help to make sure we are treating the correct condition. Personalized medicine will allow for the fabrication of personalized implants to better restore form and function after extirpative surgeries. The ability of AI to now search large medical record databases will facilitate the development of predictive models which will allow surgeons to counsel patients based on age, BMI and co-morbid disease states. This will increase predictive models of complications and allow for adjustments in post-operative care to minimize these. For medical devices, it may allow meaningful comparisons between specific devices over a large population base to show which device works the best in a specific patient.

One of the great challenges that all geographic areas face are the high costs of many of these advancements. In other areas of our economy, with technological improvements such as computers, the costs initially increase, but over time, through scaling and manufacturing efficiencies overall costs decrease. The concept of disruptive technology: faster, better and cheaper, has eluded medicine to a large degree, but as fiscal pressures become more acute, disruptive technologies may provide a way forward. The importance of publishing results in venues available to all will help us better communicate advances. As the editorial leadership at Frontiers in Surgery, we hope that this platform will help move surgery forward with many advances. We will continue to chronicle these advances and believe that global publication of these advances will help improve surgery outcomes for our patients.
